# Dysexecutive mild cognitive impairment associated to frontal atrophy: Case report

**DOI:** 10.1590/S1980-57642009DN2010001

**Published:** 2008

**Authors:** Marcio Luiz Figueredo Balthazar, Benito Pereira Damasceno

**Affiliations:** 1 MD, PhD student, Department of Neurology, Medical School, State University of Campinas, SP, Brazil.; 2 Professor, Department of Neurology, Medical School, State University of Campinas, SP, Brazil.

**Keywords:** mild cognitive impairment, executive function, Alzheimer’s disease, frontotemporal dementia, comprometimento cognitivo leve, função executiva, doença de Alzheimer, demência frontotemporal

## Abstract

Non-amnestic mild cognitive impairment (MCI) evolving to neurodegenerative diseases other than Alzheimer’s disease (AD) is rarely well documented. We report a case of a 49 year-old woman who presented a slowly progressive attentional/dysexecutive syndrome sparing other cognitive domains and without significant impairment of daily life activities. Her mother had Parkinsonism and her brother, a psychotic syndrome. Brain CT/MRI showed frontal atrophy while brain SPECT showed moderate cortical hypoperfusion, mainly in the frontal lobes. Our case is an example of non-memory MCI whose neuropsychological data and brain imaging indicating high likelihood of progression to a non-AD dementia.

## Abstract (pt)

Comprometimento cognitivo leve não-amnéstico evoluindo para uma doença neurodegenerativa outra que não doença de Alzheimer é raramente documentado. Nós descrevemos o caso de uma mulher de 49 anos de idade que apresenta uma síndrome atencional/disexecutiva lentamente progressiva, sem acometimento de outros domínios cognitivos ou comprometimento significativo de atividades de vida diária, com antecedente familiar de parkinsonismo (sua mãe) e psicose (seu irmão). Tomografia e ressonância magnética cerebral mostram atrofia frontal e o SPECT, hipoperfusão cortical moderada, principalmente em lobos frontais. Nosso caso é um exemplo de comprometimento cognitivo leve cujo perfil neuropsicológico e de neuroimagem sugere que pode haver probabilidade maior de evoluir para uma doença neurodegenerativa não-DA.

Mild cognitive impairment (MCI) is one of the most used concepts for cognitive impairment in elderly not fulfilling criteria for dementia. It can be conceived as a clinical entity for patients borderline between normal aging and very early dementia, most commonly probable Alzheimer’s disease (AD).^1^ The reporting of a decline in cognitive functioning by the patient or informant relative to previous abilities during the past year is another sign that could be helpful in identifying such patients.^3^ One of the main goals of the MCI concept is to detect individuals with high risk of developing dementia, mainly AD. As research in MCI has evolved, it has become clear that several clinical subtypes exist: amnestic MCI (single and multi-domain), and non-amnestic (single and multiple-domain).^1,3^ For example, MCI patients could present impairment in a single cognitive domain such as pronounced language disturbance evolving to primary progressive aphasia, or alteration in attentional abilities and a dysexecutive syndrome progressing to frontotemporal dementia (FTD).^4^ Very little is known about non-amnestic MCI evolving to a non-AD dementia. We report an example of non-memory MCI, in which neuropsychological data and brain imaging indicate a high likelihood of progression to a non-AD dementia.

## Case

A 49-year-old woman with eleven years of formal education came to our neuropsychological clinic with a four-year history of cognitive decline and neuropsychiatric symptoms. She noted that her performance at work (she worked with clients as a saleswoman) had gradually worsened until the point of getting fired.

The following year, in a new job, the situation worsened: she had great difficulty in learning her new job functions: what she had to do in certain situations or what paper she had to use for a promissory note, for example. The worst aspect of her poor performance was organization: she could not organize her schedule and daily activities properly. She was often late and could not fulfill her duties even after a ten-month training course. According to her, organization had always been one of her best qualities in the past but, slowly, she was losing this.

At this time, she experienced increasing difficulty with mathematical operations and was only able to perform these using calculators. Also, she was unable to retain new information given by her boss: “I listened to everything he said, but just a moment afterwards, I forgot everything and asked for further explanations”.

Due to this situation, she became depressed and used to cry even for no apparent reason, and had no pleasure in life. Following this, she underwent a psychiatric evaluation and started taking antidepressant drugs such as fluoxetin and amytriptiline, with partial improvement in mood, but not in professional performance.

**Figure 1:**
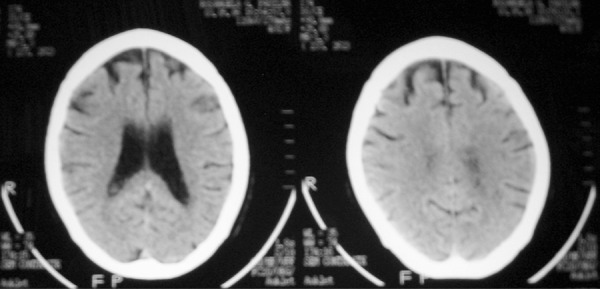
Brain CT showing frontal atrophy.

**Figure 2:**
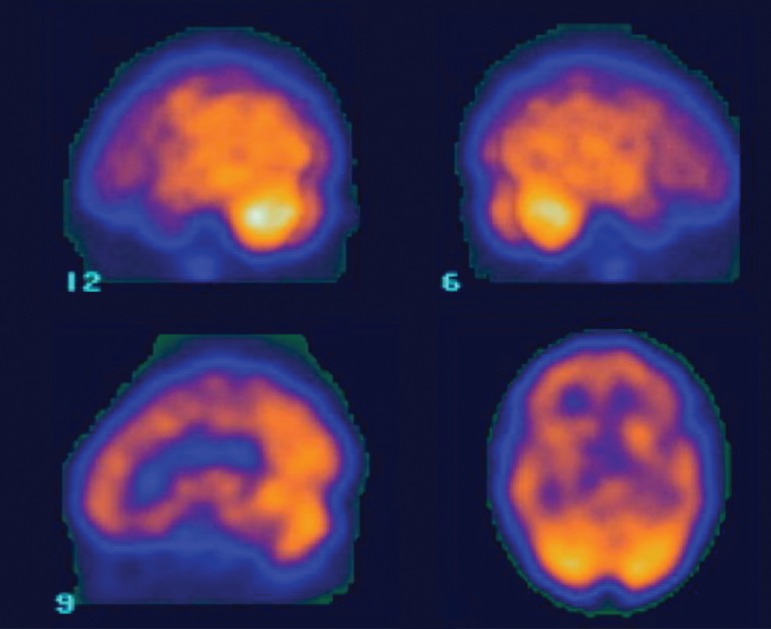
Brain SPECT showing moderate hypoperfusion in frontal lobes.

Currently, she complains of difficulty in remembering names of known people, including her family circle. She also complains of lack of concentration: with book reading, she always has to go back and reread the same page several times to understand and remember the plot of the novel. She lives with her son (who is mentally and cognitively impaired due to congenital rubeola) and does all domestic tasks satisfactorily. She is completely independent for daily living activities such as preparing meals, managing money, shopping for personal items and performing light or heavy housework. Depressive symptoms have improved with venlafaxine. Past medical history was unremarkable, except for mild hearing impairment. Her mother has a Parkinsonian syndrome, while her only brother has psychotic symptoms including persecutory delusions and aggressiveness. Physical and neurological examinations were normal. Routine laboratorial examinations including B12 and folate dosage, serology for syphilis and thyroid hormones were normal. Brain computed tomography (CT) and Magnetic Resonance Imaging (MRI) showed frontal atrophy which was disproportional for age. (Figure 1), while brain SPECT showed moderate cortical hypoperfusion, mainly to frontal lobes (Figure 2).

### Neuropsychological and psychiatric evaluation

The patient was administered the following tests: the Mini Mental Status Examination (MMSE)^5^; Luria’s Neuropsychological Investigation (LNI),^6^ subitems of memory and visuospatial perception; Stroop Test (ST);^7^ Trail Making Test (TMT)7; Boston naming Test (BNT);^7^ Frontal Assessment Battery (FAB)^8,9^ and forward and backward digit span subitems of WAIS-R^10^ whereas the Beck Depression Inventory (BDI)^11^ was used to evaluate current depression status.

The results are shown in Table 1.

**Table 1:** Neuropsychological evaluation and depression inventory.

	Scores
MMSE	25/30*
Verbal Memory (list of 10 unrelated words)	Immediate recall (mean of 10 trials): 5.7/10 Delayed recall (after 30 minutes): 7/10 Recognition: 10/10
Stroop Test	Part 1 (congruent): no errors in 92 seconds Part 2 (incongruent): 9 errors in 262 seconds
Trail Making Test A	72 seconds without errors
Trail Making Test B	180 seconds with errors^†^
Boston Naming Test (BNT)	52/60
Frontal Assessment Battery (FAB)	13/18^‡^
Forwards Digit Span	4
Backwards Digit Span	3
Verbal Fluency (category: animals)	8
Visuospatial Perception (LNI subtest)	No errors
Beck Depression Inventory	10/63

Beck Depression Inventory 10/63 MMSE: Mini-Mental Status Examination; LNI: Luria's Neuropsychological Investigation;

* She lost 1 point on recall and 4 points on calculation, but spelt "mundo" ("world") backwards correctly;

† She produced the sequence: 1A-2B-3C-4D-5E-5-6-7-F-G-8-9-G-10-H-I-13-M;

‡ Subitems: similarities: 3; lexical fluency: 1; motor series: 2; conflicting instructions: 2; inhibitory control (go-no-go): 2; prehension behavior: 3.

## Discussion

Our patient presented significant impairment mainly on tests measuring executive function (Stroop and Trail Making) and attention/short term memory (calculation on MMSE, forward and backward digit span subitems of WAIS-R) as well as verbal fluency. On the FAB, she lost points on lexical-fluency, conflicting instructions, motor series and inhibitory control (go-non-go). Episodic memory, language and visuospatial perception were unaffected. Despite the impairments, she lives alone, takes care of her son, manages money, pays bills in the bank and does all the housework. To sum up, she presents a frontal synFigure drome characterized by dysexecutive function, inattention and difficulties in inhibitory control tasks with minimally impaired daily life activities. Depressive symptoms alone cannot explain her poor performance on the tests. Brain image methods showed disproportionate frontal atrophy and hypoperfusion, unexpected for age.

For the diagnosis of dementia, ICD-10^12^ and DSM-IV^13^ criteria require significant impairment in daily life activities. Even the clinical criteria of the Work Group of Frontotemporal Dementia and Pick’s disease^14^ require that behavioral or language deficits cause significant impairment in social or occupational functioning. A drawback of the ICD-10 and DSM-IV criteria is that they focus memory impairment and do not take into account behavioral symptoms. Therefore, this patient cannot be diagnosed as demented, but it is clear she is not normal. Thus, according to the International Working Group on Mild Cognitive Impairment, she must be classified as single domain non-amnestic MCI (dysexecutive MCI).^1^

Conceptually, it is quite likely that other non-AD dementias have incipient stages where MCI terminology can accommodate the concept of this prodromal state. Even cohort studies commonly employ the classification of non-amnestic MCI – single domain, but do not specify the subtypes of non-memory domain affected.^15^ Another interesting point is the familial history of parkinsonism (her mother) and psychotic syndrome (her brother). Several neurodegenerative diseases are associated to Tau protein pathology, especially FTD plus Parkinsonism, with mutations of Tau gene (MAPT) located at chromosome 17.^16^ Moreover, mutations in the progranulin gene (PGRN) have been shown to cause familial frontotemporal lobar dementia with ubiquitinated inclusions (FTLD-U), including TDP-43 protein pathology, with variable clinical presentation such as Parkinsonism, behavioral changes and language disturbances.^17,18^ We argue whether this family could present different phenotypes that express distinct and early manifestations of Tau or TDP-43 protein pathology. Further evaluation of molecular genetics and rigorous follow-up are essential for a more definite diagnosis.
